# Factors associated with use of injectables, long-acting and permanent contraceptive methods (iLAPMs) among married women in Zambia: analysis of demographic and health surveys, 1992–2014

**DOI:** 10.1186/s12978-019-0741-6

**Published:** 2019-06-06

**Authors:** Pauline Bakibinga, Dennis Matanda, Lyagamula Kisia, Namuunda Mutombo

**Affiliations:** 1 African Population & Health Research Center, P.O.Box 10787, Nairobi, 00100 Kenya; 2 Population Council, Nairobi, Kenya; 3 University of Zambia, Lusaka, Zambia

**Keywords:** Modern contraception, Family planning, iLAPMs, Zambia

## Abstract

**Background:**

Zambia, with its women having five children on average, is one of the countries in sub-Saharan African with the highest fertility rates. As the country works on expanding its reproductive health programs, this analysis sought to understand factors behind the current utilisation of injectable, long acting and permanent methods (iLAPMs) of contraception.

**Methods:**

Cross-sectional secondary data drawn from the Zambia Demographic and Health Surveys (ZDHS) were used. This included married women aged 15–49 for the years 1992 (*n* = 620), 1996 (*n* = 1176), 2001/02 (*n* = 1483), 2007 (*n* = 1665) and 2013/14 (*n* = 4394). Frequencies, cross-tabulations and logistic regression were used to analyse levels and differentials in use of iLAPMs.

**Results:**

Except for the variables “religion” and “region”, the rest of the independent variables show significance on the use of iLAPMs, at varying levels. “Desire for children” is the strongest predictor of use of iLAPMs as it was significant at all the five data points. This is followed by “age”, although it was not significant in 2007. “Education of the woman and partner” and “number of living children” were also significant, but only for two out of the five data collection points. “Ethnicity”, “type of residence”, “heard about FP in last 12 months”, and “main decision maker on woman’s health” were only significant for one out of the five data points.

**Conclusion:**

This study has established that women’s desire for children is the main factor influencing use of iLAPMs in Zambia. Women who still want to have children are less likely to use iLAPMs even though the odds of using these methods among these women increased between 1992 and 2014. This indicates that most of this increase is due to the desire by these women to space births rather than stop having children. The 2013/2014 data also suggest an increase in access to iLAPMs among the less privileged women i.e. those in rural areas and those with low levels of education. This trend appears to have stemmed from the scaling up of family planning programmes to cover rural communities. Greater effort should be invested into family planning programs that reach all categories of women.

## Plain English summary

Sub-Saharan Africa’s (SAA) population grows rapidly each year. Many people desiring to limit childbirths still have an unmet need for family planning. Zambia is one of the countries in SAA with the highest number of children being born to one woman. On average, Zambian women have five children. As the country works on expanding its reproductive health programs, the purpose of this analysis was to understand factors behind the current utilisation of injectable, long acting and permanent methods (iLAPMs) of contraception.

We analysed data from the Zambia Demographic and Health Surveys (ZDHS). This included married women aged 15–49 for the years 1992, 1996, 2001/02, and 2013/14. The data wre analysed using different statistical analysis.

We found a strong link between the use of iLAPMs of contraception and the following factors: both maternal and partner’s educational achievement, maternal age, number of living children in a household, woman’s place of residence (urban/rural), and exposure to family planning messages through media.

Other results showed that:

- In 1996 and 2013/14, women with a primary education or no education were less likely to use iLAPMs of contraception compared to women with a secondary education or and/or a higher education.
- In 1992, 1996 and 2001/2, younger women within the ages of 15–24 years and 25–34 years, were less likely to use iLAPMs of contraception compared to women aged 35 years and older. However, in 2013 and 2014, younger women aged 15–24 were three times more likely to use iLAPMs contraception methods compared to older women. While, women aged 25–34 two times more likely.
- In 2001/2, 2007 and 20,013/4, women with 3–4 children were less likely to use iLAPMs of contraception compared to those with five or more children.
- In the 2001/2 survey, women in urban areas were twice more likely to use iLAPMs of contraception compared to residents of rural areas.

## Introduction

In 2018, the world population reached 7.6 billion people and is expected to increase by 33% to nearly 10 billion people, in 2050 [1]. Nowhere in the world is a population explosion a challenge as in sub-Saharan Africa (SSA). At approximately 3% per annum, the annual population growth rate in SSA is expected to result in 2.7 billion people by 2050. SAA together with India and China, will contribute the largest proportion to the world’s population by 2060. The decline in fertility has been substantially slower in Africa as compared to other regions like Asia, Latin America and the Caribbean at comparable stages of fertility transition [2, 3]**.** Total fertility rates in countries such as Niger (7.2), Democratic Republic of Congo (6.1), Burundi (5.7), Uganda (5.6), Nigeria (5.5), Zambia (5.0) and Tanzania (5.0) are some of the highest is SAA [4].

The current rapid annual population growth rate is largely a result of a persistently high unmet need for family planning in addition to advances in healthcare in the past two centuries that have presented the world with considerable gains in life expectancy. Nearly 200 million women (15–49 years) in developing regions who want to avoid pregnancy are not using a modern contraceptive method, with SAA having the highest proportion of women with unmet needs for modern contraception (21%) [5]**.** In developing regions, women with unmet need and using no method account for 84 and 74% respectively of all unintended pregnancies [5]**.** Two-thirds of African countries have a level of unmet needs that exceeds 25%, with East and Southern Africa having a 45% unmet need [6, 7]. Although limiting births is a major factor in driving the fertility transition and has a greater impact on fertility rates than spacing births, research has shown that only 14% of women wanted to limit child bearing compared to 25% who wanted to space births [8]. Among married women, the demand to limit was nearly equal to that for spacing [8]. Consequently, 24% of married women in Africa have an unmet need for contraception [6].

The modern contraceptive prevalence and met need has increased in SAA due to changes in behaviour consistent with ongoing family planning promotions and programmes [9]. However, none use of modern contraception remains predominant in many SAA countries despite it being more effective than traditional methods in preventing unwanted pregnancies. In East and Southern Africa alone, modern use among married women is at 36% and no method at 59% [7]. Increasing access to effective family planning methods is important because in the first year of contraceptive use, failure rates are almost zero for permanent methods, below 1.8% for long acting methods, 4.6% for short-term methods, and up to 22% for traditional methods [10]. As a result, long-acting and permanent methods (LAPMs) were found to significantly reduce the number of unintended births and induced abortions (which correlate with low access to modern contraception) if used in place of less effective methods [10, 11]. It is therefore crystal clear that the use of LAPMs offers the best strategy for countries with high fertility rates and unmet need for contraceptives [12].

Although the Zambian contraceptive prevalence rate has over the years increased from 15% (1992) to 49% (2013–14), and the use of modern contraceptives risen from 9 to 45%, over the same period, this growth has not kept pace with the annual population growth rate of 3%. On average, a woman in Zambia produces 5 children, a reduction from a total fertility rate of 6.5 reported in 1992, however, Zambian women are still having 0.8 more children than their ideal number [13]. The number of children was found to be a positively associated determinant for a woman’s total unmet need [14]. According to Pasha et al. (2015)**,** 22% of Zambian women seeking to prevent birth and 26% wanting to delay pregnancy had an unmet need [15]. Among married women, 45% were using a modern method, but 21% have an unmet need for family planning services [13]. Those with access were 19% less likely to seek family planning services with their husbands [16]. Zambia’s population is currently estimated at 16.6 million people and the proportion of women who want to limit child birth has only increased by 1% since 2007 [13]. Given the high unmet need noted above, this untamed growth has serious economic, social and health consequences as the pressure on the limited resources hinders effective delivery of vital services.

A recent analysis of the trends in modern contraceptive methods and factors related to current use in Southern Africa, including Zambia, showed that use had increased over the years and that the gap between urban and rural dwelling women had narrowed (35% vs 31%) [17]. Employment status, education level and residence were strongly associated with current use of modern contraceptives. It is important to note that not all modern contraceptive methods provide protection against unplanned and/or unwanted pregnancies.

Long-acting and permanent methods (LAPMs) are the most effective modern contraceptive methods. Their value is appreciated in their ability to reduce the incidence of unintended pregnancy, as such averting the likelihood of abortion and its complications and reducing the risk of pregnancy related deaths. In spite of their documented value, they are the least utilised methods in SSA and globally [18, 19]. The common reasons for using long-acting reversible contraception (LARC) include longer protection, better child spacing and effectiveness. There are several factors contributing to the low use of long acting and permanent methods of contraception, including method related reasons, inadequate knowledge, opposition to use, and lack of trained health providers.. For example, mothers with high knowledge of LAPMs are eight times more likely to use them than those who had no knowledge [20]**.** Moreover, Mutombo and Bakibinga, found that Zambian women who made joint decisions on contraceptives are significantly more likely to use iLAPMs than those who did not involve their husbands [21].

Barrier and short-term methods have high discontinuation and failure rates. In Zambia, 7% contraceptive users discontinued using a modern method within 12 months of starting due to side effects and/or health concerns. Women, in different studies, have also mentioned that one of the main reasons for none use is fear of side effects and/or health concerns [6, 8, 14]. Among married women other reasons include postpartum amenorrhea/breastfeeding and infrequent or no sex [6].

In 2017 the Zambian government made the Family Planning 2020 Commitment to increase the modern contraceptive prevalence rate among married women to 58% and ensure that government’s contribution to family planning commodities increases by at least 50% [22]. The services provided from 2013 to 2020 will lead to more than ten million Couple Years of Protection (CYP) [23]. With this in play, there is a need to document, extensively, the factors associated with use of long acting reversible and permanent methods, over time in Zambia. Given the importance of long acting and permanent methods and the need to provide updated information on the trends of use, this study was conceived to assess factors associated with utilization of long acting (Implant and IUCD) and permanent (Vasectomy and Female sterilization) contraceptive methods among married women of reproductive age (15–49 years) in Zambia.

The current international development agenda, under the Sustainable Development Goals (SDG) umbrella stresses the need to ensure equitable access to services for all, including, health, but especially for those in most need. This assertion comes in the wake of concerns about reduced funding for family planning programs in developing countries. As countries experiencing huge population explosions try to expand their reproductive health programs, it is important that attention is given to the factors for and against universal access to services. This analysis was conducted with a desire to understand what has been and is behind the current utilisation of long acting and permanent methods of contraception. This is important as it enables interrogation of the use of these effective methods in a context where many interventions geared at enhancing universal health care, including family planning programs, have been implemented.

## Methods

### Data source

**Fig. 1:**
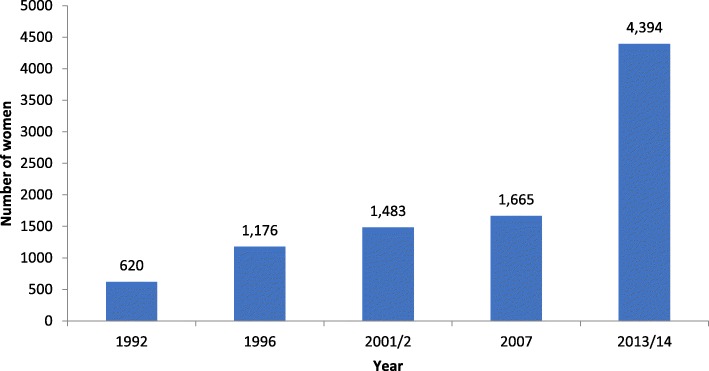
Number of eligible women for analysis, ZDHS 1992–2014

The data for this paper were drawn from the women files from the Zambia Demographic and Health Surveys (ZDHS) for the years 1992, 1996, 2001/2, 2007, and 2013/14. The methodology used in these surveys has been comprehensively described in the respective ZDHS reports by the Zambia Central Statistical Office [13, 24–27]. The main objective of these surveys was to provide information on levels and trends in fertility, childhood mortality, use of family planning methods, and maternal and child health indicators including HIV/AIDS. These are nationally representative surveys whose sample design is tailored to provide specific indicators at national and provincial levels. The sampling frames for the 1992 and 1996 surveys were based on 1990 Zambia Census of Population and Housing (ZCPH), while the 2001/2 and 2007 surveys were based on the 2000 ZCPH. The 2013/14 survey was based on the 2010 ZCPH. Over the years, the sample size for these surveys has increased and so is the size of the eligible women for our analysis. Figure 1 shows that the sample of eligible women (married women aged 15–49) rose by more than 6 times between 1992 and 2013/14.

### Variables

The analytical models used in this paper are adapted from the 2014 paper by Mutombo and Bakibinga, which analysed the effect of joint contraceptive decisions on the use of iLAPMs in Zambia [21]. The dependent variable used is type of contraceptive method. This variable is measured as a dichotomous variable and is coded as short-acting and long-acting. Short acting include all traditional and folkloric methods as well as modern methods that require some action on the part of the user just before sexual intercourse or on daily basis (e.g. condom, spermicides, oral pill). On the other hand, long-acting methods comprise of injectable contraceptives, implants and permanent methods. Our paper uses 11 independent variables based on earlier works by Mutombo and Bakibinga [21]. These variables are: “age”, “number of living children”, “ethnicity”, “desire for children”, “heard about family planning (FP) in last 12 months”, “religion”, “type of residence”, “respondent’s level of education”, “partner’s level of education”, “region”,, and ***“***main decision maker on woman’s health”.

Both “level of education of respondent” and “level of education of partner” are used rather than level of education for the respondent only, as partner education could have some influence on women’s decisions [28]. In order to assess some power relations between women and men regarding health issues (contraceptive choices), we used the variable “main decision maker on woman’s health”. However, information on power relations was not collected for the 1992 and 1997 surveys.

The variable “region” was constructed by grouping the ten provinces into two groups. The grouping was based on proximity to the main line of rail, which to some extent defines access to various services in Zambia. Region 1 comprises of the four provinces (Central, Copperbelt, Lusaka and Southern) where the main development corridor lies. Region 2 covers the other six provinces (Eastern, Luapula, Muchinga, Northern, North Western and Western) outside the main development corridor .

### Data analysis

In order to ascertain the main factors associated with use of iLAPMs the study used different teachniques in analysing the data. Frequencies, cross-tabulations and logistic regressions were used to analyse levels and differentials in use of long-acting family planning methods among women (15–49) in Zambia. The logit of the logistic regression model used can be expressed as follows [29]:

$$ {\displaystyle \begin{array}{c}g\left(\mathrm{x}\right)=\ln \left[\frac{\pi (x)}{1-\pi (x)}\right]\\ {}=\left[\pi (x)/1-\pi (x)\right]\\ {}={\beta}_0+{\beta}_1{x}_1+{\beta}_2{x}_2+\dots +{\beta}_{\mathrm{p}}{x}_{\mathrm{p}},\end{array}} $$

Where:

$$ {\displaystyle \begin{array}{l}\pi \kern0.24em (x)\kern1.2em \mathrm{the}\ \mathrm{odds}\ \mathrm{for}\ \mathrm{the}\ \mathrm{dependent}\ \mathrm{variable}\\ {}{x}_1.\dots {x}_{\mathrm{p}}\kern0.72em \mathrm{independent}\ \mathrm{variable}\mathrm{s}\\ {}{\beta}_0\kern1.8em \mathrm{constant}\\ {}{\beta}_1\dots {\beta}_{\mathrm{p}}\kern0.84em \mathrm{regression}\ \mathrm{coefficients}\end{array}} $$

The odds ratio is used to depict the likelihood of using LAPM. A ratio greater than 1 means greater likelihood than the reference category; a ratio less than 1 means lower likelihood than the reference category; and a ratio of 1 means same likelihood as the reference category. In this study, an independent variable is considered significant if its effect on the dependent variable (type of contraceptive method) is statistically significant at the .95 confidence interval (i.e. *p* ≤ 0.050). Given the multi-stage sampling strategy employed by DHS, all analyses were weighted and missing data handled by pairwise deletion.

## Results

### Sample characteristics

Distribution of married women aged 15–49 by selected background characteristics from 1992 to 2013/4 is shown in Table 1. Majority of women sampled across the five surveys had primary level of education while their partners had secondary and/or higher education. Majority of women interviewed were aged 25–34, had at least five or more children, and desired for more children. In terms of ethnicity, women of Bemba extraction formed the largest proportion of the sampled population. Most of the women interviewed were non-Catholic, were living in rural areas, and had heard about FP in the media during the last 12 months prior to respective surveys. In the earlier survey (2001/2), majority of sampled women had decision on their health made by other people than by the woman herself or in consultation with the partner. However, this changed in the subsequent surveys (2007 and 2013/4) where decision on a woman’s health was majorly made by the woman and the partner other than solely by the woman or by other people.

**Table 1:** Percent distribution of married women aged 15–49 by selected background characteristics: 1992–2014

Background characteristics	1992		1996		2001/2		2007		2013/4	
	%	*n*	%	*n*	%	*n*	%	*n*	%	*n*
*Level of education*										
None	11.3	70	12.8	150	10.6	157	10.5	175	8.2	361
Primary	53.7	333	58.3	686	56.9	844	55.3	921	52.2	2294
Secondary+	35.0	217	28.9	340	32.5	482	34.2	569	39.6	1739
*Partner’s level of education*										
None	5.6	35	6.3	74	4.6	68	5.0	84	4.8	213
Primary	38.7	240	41.8	491	43.0	638	42.7	711	36.6	1608
Secondary+	55.7	345	51.9	611	52.4	777	52.2	870	58.6	2573
*Age*										
15–24	26.8	166	30.7	361	29.0	430	25.3	421	21.4	939
25–34	42.2	262	40.4	475	43.2	640	46.8	780	45.4	1995
35+	31.0	192	28.9	340	27.8	413	27.9	464	33.2	1460
*Number of living children*										
≤ 2	30.8	191	33.5	394	34.4	511	33.4	556	30.8	1353
3–4	24.8	154	29.2	343	31.5	467	35.6	592	33.8	1483
5+	44.4	275	37.3	439	34.1	505	31.0	517	35.4	1558
*Desire for children*										
Wants more	56.9	353	60.7	714	54.9	814	55.6	925	56.1	2464
Undecided/wants no more	43.1	267	39.3	462	45.1	669	44.4	740	43.9	1930
*Ethnicity/language group*										
Bemba	43.5	270	36.1	424	42.7	633	34.4	572	42.2	1854
Lozi	6.6	41	6.2	73	6.4	95	8.5	141	5.8	255
North Western (Kaonde, Lunda & Luvale)	12.7	79	17.8	210	13.2	196	11.4	190	10.0	439
Nyanja	21.9	136	24.4	287	23.0	341	30.2	503	26.1	1148
Tonga	15.3	94	15.5	182	14.7	218	15.5	259	15.9	698
*Religion/Denomination*										
Catholic	29.5	183	24.0	282	23.9	355	19.2	319	17.7	778
Non-Catholic	70.5	437	76.0	894	76.1	1128	80.8	1346	82.3	3616
*Region*										
Region 1 (Central, Copperbelt, Lusaka & Southern)	63.7	395	48.6	571	48.5	719	48.2	802	43.6	1914
Region 2 (Eastern, Luapula, Muchinga, Northern, North Western & Western)	36.3	225	51.4	605	51.5	764	51.8	863	56.4	2480
*Type of residence*										
Urban	58.9	365	44.0	517	40.4	600	44.4	740	47.7	2098
Rural	41.1	255	56.0	659	59.6	883	55.6	925	52.3	2296
*Heard about FP in the media during last 12 months*										
Yes	29.8	185	63.2	743	57.6	855	50.8	846	43.3	1903
No	70.2	435	36.8	433	42.4	628	49.2	819	56.7	2491
*Main decision maker on woman’s health*										
Woman alone	–	–	–	–	31.1	461	31.1	517	31.6	1386
Woman with partner	–	–	–	–	11.7	173	36.7	611	43.7	1921
Other	–	–	–	–	57.2	849	32.2	537	24.7	1087
Total	100.0	620	100.0	1176	100.0	1483	100.0	1665	100.0	4394

### Distribution of iLAPMs users by selected background characteristics from 1992 to 2013/4

Table 2 is a summary of the distribution of contraceptive users by type of FP method and selected background characteristics. Nationally, apart from the decline in the use of iLAPMs in the early years (18% in 1992 and 14% in 1996), Zambia experienced a marked increase in the use of iLAPMs from 1996 to 2013/14 (14% in 1996, 20% in 2001/2, 27% in 2007 and 57% in 2013/4). Nonetheless, national trends can mask significant differences in the use of iLAPMs by sociodemographic segments. Across all or four out of the five surveys, there were statistically significant differences in the proportion of women who used iLAPMs by their level of education, partner’s level of education, maternal age, number of living children, region of residence, type of residence (urban/rural) and exposure to FP messages through media. There were statistically significant differences in the proportion of women who used iLAPMs in two out of the three surveys where data on decision making on a woman’s health was available.

**Table 2:** Percent distribution of contraceptive users by type of FP method and selected background characteristics: 1992–2014

Background characteristics	Type of FP method									
	1992		1996		2001/2		2007		2013/4	
	iLAPMs	Other	iLAPMs	Other	iLAPMs	Other	iLAPMs	Other	iLAPMs	Other
*Level of education*	**	**	***	***	***	***	**	**	*	*
None	17.4	82.6	8.0	92.0	13.0	87.0	20.4	79.6	52.2	47.8
Primary	13.6	86.4	11.1	88.9	17.9	82.1	25.0	75.0	58.8	41.2
Secondary+	23.9	76.1	20.3	79.7	25.5	74.5	31.1	68.9	55.8	44.2
*Partner’s level of education*	*	*	**	**	***	***	***	***	*	*
None	31.2	68.8	9.5	90.5	4.7	95.3	20.0	80.0	64.8	35.2
Primary	12.8	87.2	10.2	89.8	16.1	83.9	22.0	78.0	56.1	43.9
Secondary+	19.9	80.1	16.1	46.8	24.1	75.9	30.8	69.2	56.5	43.5
*Age*	***	***	***	***	***	***	**	**	***	***
15–24	1.7	98.3	3.1	96.9	9.7	90.3	25.1	74.9	65.2	34.8
25–34	10.0	90.0	8.0	92.0	18.5	81.5	23.3	76.7	56.8	43.2
35+	40.9	59.1	32.4	67.6	33.5	66.5	32.6	67.4	52.2	47.8
*Number of living children*	***	***	***	***	***	***	**	**		
≤ 2	10.8	89.2	5.6	94.4	12.9	87.1	25.0	75.0	57.7	42.3
3–4	11.6	88.4	12.0	88.0	16.7	83.3	23.0	77.0	55.1	44.9
5+	26.6	73.4	22.7	77.3	30.9	69.1	32.0	68.0	58.3	41.7
*Desire for children*	***	***	***	***	***	***	***	***		
Wants more	3.5	96.5	3.9	96.1	10.6	89.4	22.9	77.1	56.4	43.6
Undecided/wants no more	35.5	64.5	27.9	72.1	31.7	68.3	30.8	69.2	58.1	41.9
*Ethnicity/language group*									**	**
Bemba	17.7	82.3	14.7	85.3	19.8	80.2	26.8	73.2	56.9	43.1
Lozi	17.1	82.9	9.4	90.6	17.0	83.0	20.4	79.6	55.3	44.7
North Western (Kaonde, Lunda & Luvale)	19.7	80.3	12.3	87.7	20.0	80.0	34.8	65.2	65.1	34.9
Nyanja	12.7	87.3	12.0	88.0	20.6	79.4	23.9	76.1	58.5	41.5
Tonga	16.8	83.2	14.7	85.3	23.0	77.0	28.2	71.8	53.5	46.6
*Religion/Denomination*							*	*		
Catholic	18.9	81.1	11.0	89.0	17.4	82.6	21.7	78.3	58.3	41.7
Non-Catholic	17.5	82.5	14.6	85.4	20.9	60.1	27.6	72.4	56.8	43.2
*Region*	**	**	***	***	***	***	**	**	**	**
Region 1 (Central, Copperbelt, Lusaka & Southern)	20.5	79.5	18.7	81.3	24.0	76.0	29.1	70.9	55.3	44.7
Region 2 (Eastern, Luapula, Muchinga, Northern, North Western & Western)	11.7	88.3	6.8	93.2	14.6	85.4	23.3	76.7	59.7	40.3
*Type of residence*	***	***	***	***	***	***	**	**	**	**
Urban	22.1	77.9	19.5	80.5	26.7	73.3	30.7	69.3	54.8	45.2
Rural	10.0	90.0	7.5	92.5	14.2	85.8	23.4	76.6	59.0	41.0
*Heard about FP in the media during last 12 months*			**	**	***	***	**	**	***	***
Yes	20.0	80.0	16.0	84.0	24.2	75.8	29.5	70.5	53.4	46.6
No	16.8	83.2	9.2	90.8	13.5	86.5	23.4	76.6	59.6	40.4
*Main decision maker on woman’s health*					**	**	**	**		
Woman alone	–	–	–	–	24.3	75.7	27.5	72.5	56.0	44.0
Woman with partner	–	–	–	–	22.5	77.5	30.4	69.6	57.1	42.9
Other	–	–	–	–	17.3	82.7	21.2	78.8	58.3	41.7
Total	17.9	82.1	13.7	86.3	20.3	79.7	26.5	73.5	57.2	42.8

*, *p* < .050; **, *p* < .010; ***, *p* < .001

To begin with, mothers with secondary and /or higher education were the highest users of iLAPMs in 1992, 1996, 2001/2 and 2007. However, the trend changed in 2013/4 where mothers with primary education were the highest users of iLAPMs compared to mothers in the other educational categories. Trends in the use of iLAPMs by partner’s level of education indicate a consistently higher use among those with secondary and/or higher education from 1996 to 2007. In contrast, surveys conducted in 1992 and 2013/4 showed higher use of iLAPMs among partners with no education.

Use of iLAPMs by maternal age showed higher use among women aged 35 years and older in surveys conducted in 1992, 1996, 2001/2 and 2007. The pattern however changed in the 2013/4 survey whereby younger women aged 15–24 posted the highest use of iLAPMs compared to women in the other age groups. Patterns of iLAPMs use disaggregated by number of living children and mother’s desire for children showed that women with five or more children and those who were either undecided or wanted no more children were the highest users of iLAPMs in 1992, 1996, 2001/2 and 2007.

Regarding place of residence, women living in Region 1 and urban areas posted higher prevalence in the use of iLAPMs from 1992 to 2007. The pattern was however reversed in 2013/4 whereby women living in Region 2 and rural areas posted significantly higher prevalence in the use of iLAPMs. Results on the use of iLAPMs by exposure to FP messages through media showed in 1996, 2001/2 and 2007, women who had heard about FP in the media during the last 12 months prior to the survey constituted the largest proportion of iLAPMs users compared to those who did not hear about FP in the media. The opposite was nonetheless true in the 2013/4 survey whereby women who had not heard about FP in the media were the highest iLAPMs users compared to those who had heard about FP in the media. In 2001/2 and 2007, women whose decision on their health was made by other people had significantly lower use of iLAPMs as compared to women who either made such a decision on their own or with their partners. The differences in iLAPMs use by decision making on a woman’s health was nonetheless not statistically significant in 2013/4.

### Likelihood of married women using iLAPMs from 1992 to 2013/4

Table 3 shows unadjusted and adjusted logistic regression models assessing the likelihood of women using iLAPMs in 1992, 1996, 2001/2, 2007 and 2013/4. Beginning with unadjusted models, in four out of the five surveys, there were statistically significant associations between the use of iLAPMs and maternal and partner’s level of education, maternal age, number of living children, desire for children, region of residence, type of residence (urban/rural), and exposure to FP messages through media. There was a statistically significant association between the use of iLAPMs and decision making on a woman’s health in 2007 only as this variable was insignificant on the use of iLAPMs in 2001/22013/4. In 2007, women who made decisions on their health alone ((AOR = 1.394, 95% CI 1.044–1.852) and those who made such decisions in consultation with their partners (AOR = 1.506, 95% CI 1.139–1.992) were more likely to use iLAPMs compared to women whose decision on their health was made by other people.

**Table 3:** Likelihood of using iLAPMs among married female contraceptive users by selected background characteristics: 1992–2014

Background characteristics	Unadjusted and Adjusted Odds ratios									
	1992		1996		2001/2		2007		2013/4	
	OR	AOR	OR	AOR	OR	AOR	OR	AOR	OR	AOR
*Respondent’s level of education*										
None	0.663	0.709	0.325**	0.411*	0.438**	0.695	0.564**	0.709	0.867	0.709**
Primary	0.502**	1.010	0.488***	0.544*	0.638**	0.869	0.737**	0.885	1.130*	0.985
Secondary+^†^	–	–	–	–	–	–	–	–	–	–
*Partner’s level of education*										
None	1.736	3.070	0.546	0.959	0.154**	0.226*	0.556*	0.689	1.418*	1.272
Primary	0.599*	0.751	0.590**	0.839	0.604***	0.844	0.632***	0.731*	0.983	0.915
Secondary+	–	–	–	–	–	–	–	–	–	–
*Age*										
15–24	0.028***	0.047***	0.069***	0.132***	0.214***	0.455**	0.967*	1.046	1.716***	3.212***
25–34	0.161***	0.189***	0.183***	0.213***	0.449***	0.618**	0.631***	0.852	1.206**	1.771***
35 + ^†^	–	–	–	–	–	–	–	–	–	–
*Number of living children*										
≤ 2	0.330***	2.401	0.199***	1.307	0.333***	0.763	0.710**	0.745	0.976	0.594***
3–4	0.360***	1.024	0.460***	1.517	0.451***	0.671*	0.637**	0.730	0.877	0.713***
5 + ^†^	–	–	–	–	–	–	–	–	–	–
*Desire for children*										
Wants more	0.066***	0.182***	0.105***	0.238***	0.255***	0.440***	0.667***	0.755*	0.936	0.695***
Undecided/wants no more^†^	–	–	–	–	–	–	–	–	–	–
*Ethnicity/language group*										
Bemba	1.068	1.173	1.004	0.868	0.823	0.955	0.931	0.878	1.150	1.160
Lozi	1.059	1.749	0.618	0.561	0.693	0.891	0.640	0.595	1.074	1.104
North Western (Kaonde, Lunda & Luvale)	1.251	2.152	0.834	1.452	0.848	1.097	1.361	1.312	1.627***	1.753***
Nyanja	0.703	0.560	0.811	1.105	0.864	0.978	0.793	0.898	1.230*	1.273*
Tonga^†^	–	–	–	–	–	–	–	–	–	–
*Religion/Denomination*										
Catholic	1.084	1.150	0.733	0.702	0.799	0.779	0.728	0.813	1.067	1.027
Non-Catholic^†^	–	–	–	–	–	–	–	–	–	–
*Region*										
Region 1 (Central, Copperbelt, Lusaka & Southern)	1.967**	1.099	3.124***	1.685	1.838***	1.230	1.353**	0.963	0.833**	0.979
Region 2 (Eastrn, Luapula, Muchinga, Northern, North Western & Western) ^†^	–	–	–	–	–	–	–	–	–	–
*Type of residence*										
Urban	2.548***	2.096	2.956***	1.625	2.212***	1.537*	1.453**	1.119	0.841**	0.892
Rural^†^	–	–	–	–	–	–	–	–	–	–
*Heard about FP in the media during last 12 months*										
Yes	1.241	1.151	1.879**	1.016	2.060***	1.258	1.366**	1.124	0.776***	0.814**
No^†^	–	–	–	–	–	–	–	–	–	–
*Main decision maker on woman’s health*										
Woman alone	–	–	–	–	1.523**	1.270	1.411*	1.390*	0.905	0.963
Woman with partner	–	–	–	–	1.371	1.416	1.628***	1.506**	0.948	1.010
Other^†^	–	–	–	–	–	–	–	–	–	–
Constant	**–**	0.420	**–**	0.501	**–**	0.469*	**–**	0.558*	**–**	1.355*

*, *p* < .050; **, *p* < .010; ***, *p* < .001; *OR* Unadjusted Odds Ratios, *AOR* Adjusted Odds Ratios; +, and Higher; †, Reference Group

In the adjusted models, maternal education was significantly associated with iLAPMs use only in the 1996 and 2013/4 surveys. In 1996, women with primary (AOR = 0.544, 95% CI 0.342–0.866) or no education (AOR = 0.411, 95% CI 0.173–0.973) were less likely to use iLAPMs compared to women with secondary and/or higher education. Similarly, in 2013/4, women with no education were less likely to use iLAPMs (AOR = 0.709, 95% CI 0.548–0.918) compared to women with secondary and/or higher education. Partner’s level of education was significantly associated with the likelihood of iLAPMs use only in the 2001/2 and 2007 surveys. Women whose partners had no education in 2001/2 (AOR = 0.226, 95% CI 0.066–0.772) or had primary level education in 2007 (AOR = 0.731, 95% CI 0.560–0.954) were significantly less likely to use iLAPMs compared to women with partners with secondary and/or higher education.

Controlling for other covariates in the regression model, age was significantly associated with iLAPMs use in four out of the five surveys. In 1992 (AOR = 0.047, 95% CI 0.011–0.198; AOR = 0.189, 95% CI 0.098–0.364), 1996 (AOR 0.132, 95% CI 0.055–0.313; AOR 0.213, 95% CI 0.128–0.356) and 2001/2 (AOR = 0.455, 95% CI 0.258–0.804; AOR = 0.618, 95% CI 0.431–0.885), younger women aged 15–24 and 25–34 were less likely to use iLAPMs compared to women aged 35 years and older. The pattern was however different in 2013/4 where younger women aged 15–24 (AOR = 3.212, 95% CI 2.515–4.103) and 25–34 (AOR = 1.771, 95% CI 1.493–2.101) were three and two times, respectively, likely to use iLAPMs compared to women aged 35 years and older.

Number of living children in a household was significantly associated with the likelihood of women using iLAPMs in 2001/2 and 2013/4. In 2001/2 and 20,013/4, women with 3–4 children ((AOR = 0.671, 95% CI 0.462–0.976) and (AOR = 0.713, 95% CI 0.600–0.846), respectively) were less likely to use iLAPMs compared to those with five or more children. Equally, women with two or less children ((AOR = 0.594, 95% CI 0.474–0.744)) were significantly less likely to use iLAPMs in 2013/4 compared to those with five or more children. Mother’s desire for children was significantly associated with iLAPMs use in all the surveys. Women who wanted more children were significantly less likely to use iLAPMs in 1992 (AOR = 0.182 95% CI 0.087–0.380), 1996 (AOR = 0.238 95% CI0.138–0.408), 2001/2 (AOR = 0.440, 95% CI 0.312–0.621), 2007 (AOR = 0.755, 95% CI 0.571–0.999), and 2013/4 (AOR = 0.695, 95% CI 0.595–0.812).

Considering other possible covariates of iLAPMs use, a woman’s place of residence was significantly associated with iLAPMs use only in the 2001/2 survey. Women in urban areas (AOR = 1.537, 95% CI 1.088–2.172) were two times morelikely to use iLAPMs compared to women residing in rural areas. The Zambian data also show some interesting trends on the significance of exposure to FP messages on the use of iLAPMs. Regarding access to FP messages, the data show that while a woman’s exposure to FP messages in last 12 months was not significant on the use of iLAPMS in the prior surveys, the data for the 2013/4 survey was significantly associated with the likelihood of using iLAPMs. Women who had heard about FP in during the 12 months to their interview (AOR = 0.814, 95% CI 0.717–0.924) were less likely to use iLAPMs compared to those who had not heard about FP during the reference period. A similar trend is also notable by ethnicity. Whereas earlier surveys show no significance on the use of iLAPMS by ethnicity, the 2013/14 survey reveals significant differences among ethnic groups. While the Bemba and Lozi women exhibit higher likelihood of using iLAPMs than their Tonga counterparts (reference group), the relationship is statistically insignificant. On the other hand, women of North Western ethnicities (Kaonde, Lunda and Luvale) (AOR = 1.753, 95% CI 1.310–2.345) and those of Nyanja-Speaking groups (AOR = 1.273, 95% CI 1.039–1.559) are significantly more likely to use iLAPMs than their Tonga counterparts, with North Westerners almost twice more likely to use iLAPMs than Tonga women.

## Discussion

This paper explored the factors associated with use of injectables, long acting and permanent methods of contraception among married women in Zambia. Over the years, the use of iLAPMs has significantly increased, which can have a positive influence on the socio-economic development of countries [30]. However, there is need for more research to understand the dynamics of contraceptive use among Zambian women as trends appear inconsistent. These trends, point to issues of access among the vulnerable groups. By 1996 (five years after country reverted to multi-party democracy), Zambia had stabilised economically with a better functioning health system than the situation in 1992 (a year after the change of government). By 2001/2, the country was struggling with a huge external debt that affected health service delivery (including family planning) in a number of ways. In 2007, the country had just gotten debt relief from the HIPC initiative and the effects of this relief had not fully trickled to most parts of the country. The 2013/14 results show much higher access to iLAPMs than previously, possibly owing to transfer of certain services from the Ministry of Health to the Ministry of Community Development in 2011. With a well performing economy between 2006 and 2011, the government was able to roll out a range of health services (including family planning) to the most vulnerable groups through static and mobile health services.

Data show that use of modern contraceptives in Zambia has been significantly associated with employment status, education level and residence, as elsewhere. For instance, results from different regions in Ethiopia found that women’s level of education, number of live children, occupation, and discussions with health care providers were significantly positively associated with the use of LAPMs [31–33]**.** In this study, maternal education was found to be significantly associated with iLAPMs use only in the 1996 and 2013/4 surveys. In both surveys, women with primary or no education were less likely to use iLAPMs compared to women with secondary and/or higher education. Similarly, it has also been reported that utilisation of iLAPMs were significantly associated with women who had secondary school, college and above education [34]. Partner’s level of education significantly predicted the likelihood of iLAPMs use only in the 2001/2 and 2007 surveys. Women with partners with no education in 2001/2 or had primary level of education in 2007 were significantly less likely to use iLAPMs compared to women with partners with secondary and/or higher education.

Maternal age was a significantly associated with iLAPMs use in four out of the five surveys after controlling for other factors in the regression model. In the earlier surveys 1992, 1996 and 2001/2, younger women aged 15–24 and 25–34 were less likely to use iLAPMs compared to women aged 35 years and older. However, in 2013/4 where younger women aged 15–24 and 25–34 were three and two times, respectively, more likely to use iLAPMs compared to women aged 35 years and older. This could be explained by younger women wanting to have less children. The 2014 Zambia Demographic and Health Survey reported that the ideal number of children for women 15–34 was less than those above 35 [13]. This is an important finding as it speaks to the younger generation and the possibility that efforts to reach young people to use ILAPMs is bearing fruits in Zambia.

Additionally, the desire to limit child bearing increases with the number of living children [13]. The number of living children in a household was significantly associated with the likelihood of women using iLAPMs in 2001/2, 2007 and 2013/4. In 2001/2, 2007 and 20,013/4, women with 3–4 children) were less likely to use iLAPMs compared to those with five or more children. Equally, women with two or less children were less likely to use iLAPMs in 2013/4 compared to those with five or more children. Additionally, women who wanted more children were significantly less likely to use iLAPMs as compared to those who were undecided or wanted no more across all the surveys. According to Van Lith et al. women who have met or exceeded their ideal parity are more likely to use permanent methods [8].

Considering other possible predictors of iLAPMs use, a woman’s place of residence was a significant covariate of iLAPMs use only in the 2001/2 survey. Women in urban areas were two times more likely to use iLAPMs compared to women residing in rural areas. This is consistent with what has been reported in the literature as women living in urban areas are likely to have better access to family planning services [35]. Important to note is the lack of statistically significant differences between urban and rural areas in the later surveys. It looks like in the early years (2002 and before) iLAPMs use in rural areas was significantly lower as compared to urban. The lack of any significant differences in the later surveys could mean there has been an increase in iLAPMs use in rural areas and the gap between urban and rural areas has significantly narrowed in the recent past.

Woman’s exposure to FP messages 12 months prior to the 2013/4 survey was significantly associated with the likelihood of iLAPMs use. Women who had heard about FP were less likely to use iLAPMs compared to those who had not heard about FP in the last 12 months. This is a new trend in Zambia and is inconsistent with Melka et al. (2015) who found that having access to media platforms like radio/TV were positively associated with the use of iLAPMs [33]. A possible explanation would be the age at which women get exposed to messages about family planning. Weidert et al. [36] reported that women 15–24 years were more likely to use contraception if they had a FP message, but there was no association with contraceptive use in older women. It can also be argued that given the nature of iLAPMs, particularly the long-acting and permanent methods, a woman who heard the message about FP more than 12 months before the survey may still be on any of these methods even when they did not hear about FP during the reference period at the time of the survey.

Another new trend is the significance of ethnicity on use of iLAPMs. It is not immediately clear why women of North Western ethnicities (Kaonde, Lunda and Luvale) and those of Nyanja-Speaking groups are significantly more likely to use iLAPMs than their Tonga counterparts. It would appear that there are some underlying cultural myths/beliefs about FP in general and effects of iLAPMS in particular within socio-ethnic networks. Understanding this complex issue would require a separate investigation.

## Conclusion

This study has established that women’s desire for children is the main factor influencing use of iLAPMs in Zambia. Women who still want to have children are less likely to use iLAPMs even though the odds of using these methods among these women increased between 1992 and 2014. This indicates that most of this increase is due to the desire by these women to space births rather than stop having children. The 2013/2014 data also suggest an increase in access to iLAPMs among the less privileged women i.e. those in rural areas and those with low levels of education. This trend appears to have stemmed from the scaling up of family planning programmes to cover rural communities. Demystifying family planning through culturally appropriate messaging and targeting women in the immediate postpartum period could be powerful tools in increasing acceptability of iLAPMSs in the country. If Zambia is to achieve the FP2020 target, greater effort should be invested into family planning programs that will empower women to be able to make informed decisions about the contraceptive methods that they use. As Zambia and other countries in SSA develop their strategies to achieve universal health access, under the SDG agenda, it is important to take stock of what influences use of these very effective methods over time and which factors can be enhanced to ensure that no woman is left behind.

## Data Availability

Data used in this study is publicly available and can be accessed through application to MEASURE DHS. Syntaxes and outputs generated can be made available upon request to the corresponding author.
